# Reduced Auditory Mismatch Negativity Reflects Impaired Deviance Detection in Schizophrenia

**DOI:** 10.1093/schbul/sbaa006

**Published:** 2020-02-19

**Authors:** Daisuke Koshiyama, Kenji Kirihara, Mariko Tada, Tatsuya Nagai, Mao Fujioka, Kaori Usui, Tsuyoshi Araki, Kiyoto Kasai

**Affiliations:** 1 Department of Neuropsychiatry, Graduate School of Medicine, The University of Tokyo, Tokyo, Japan; 2 The International Research Center for Neurointelligence (WPI-IRCN) at The University of Tokyo Institutes for Advanced Study (UTIAS), The University of Tokyo, Tokyo, Japan; 3 Department of Psychiatry, Kawamuro Memorial Hospital, Niigata, Japan

**Keywords:** mismatch negativity (MMN), deviance detection, adaptation, schizophrenia, translatable physiological biomarker

## Abstract

The auditory mismatch negativity (MMN) is a translatable electroencephalographic biomarker automatically evoked in response to unattended sounds that is robustly associated with cognitive and psychosocial disability in patients with schizophrenia. Although recent animal studies have tried to clarify the neural substrates of the MMN, the nature of schizophrenia-related deficits is unknown. In this study, we applied a novel paradigm developed from translational animal model studies to carefully deconstruct the constituent neurophysiological processes underlying MMN generation. Patients with schizophrenia (*N* = 25) and healthy comparison subjects (HCS; *N* = 27) underwent MMN testing using both a conventional auditory oddball paradigm and a “many-standards paradigm” that was specifically developed to deconstruct the subcomponent adaptation and deviance detection processes that are presumed to underlie the MMN. Using a conventional oddball paradigm, patients with schizophrenia exhibited large effect size deficits of both duration and frequency MMN, consistent with many previous studies. Furthermore, patients with schizophrenia showed selective impairments in deviance detection but no impairment in adaptation to repeated tones. These findings support the use of the many-standards paradigm for deconstructing the constituent processes underlying the MMN, with implications for the use of these translational measures to accelerate the development of new treatments that target perceptual and cognitive impairments in schizophrenia and related disorders.

## Introduction

Patients with schizophrenia exhibit impairments in early auditory information processing (EAIP) that are highly associated with cognitive and psychosocial functional disability.^1–6^ The mismatch negativity (MMN) is a translatable neurophysiological event-related potential measure of EAIP that has been widely used in the study of patients with schizophrenia and as a sensitive biomarker of responses to pharmacological and nonpharmacological challenges.^7–9^ The MMN is typically assessed using a passive auditory oddball paradigm in response to unattended stimuli that occasionally differ from frequently presented stimuli in some physical characteristics (eg, duration: dMMN; or pitch/frequency: fMMN). A reduced MMN amplitude may reflect N-methyl-d-aspartate (NMDA) receptor dysfunction in patients with schizophrenia because NMDA-R antagonists reduce the MMN amplitude.^10,11^ Prior studies showed that ketamine, an antagonist of NMDA receptors, induces schizophrenia-like symptoms in healthy volunteers.^12,13^ MMN-like responses have been demonstrated in various animals, including mice, rats, and nonhuman primates.^14–17^ Thus, it may be possible to investigate the neural mechanisms underlying the MMN in animal studies for translation to clinical settings, which might facilitate better treatments of schizophrenia.

**Fig. 1.**
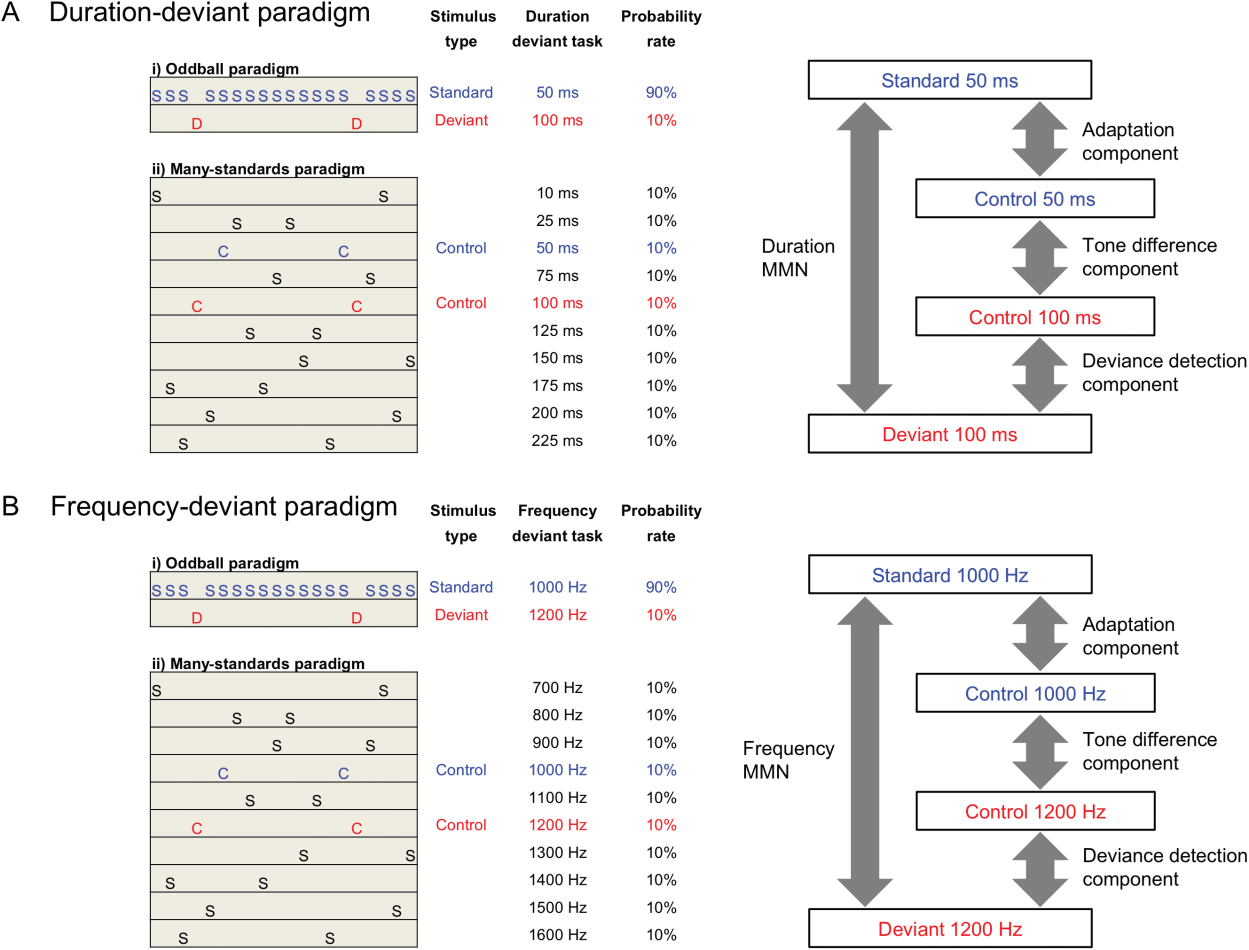
Oddball paradigm, many-standards paradigm, and components of mismatch negativity (MMN). (**A**) A 2-tone auditory oddball paradigm was performed to evaluate the duration MMN. The many-standards paradigm, as a control task for the oddball paradigm, was performed to evaluate adaptation, tone difference, and deviance detection components of the duration MMN. (**B**) A 2-tone auditory oddball paradigm was performed to evaluate the frequency MMN. The many-standards paradigm, as a control task for the oddball paradigm, was performed to evaluate adaptation, tone difference, and deviance-detection components of the frequency MMN (Colored figure is available online).

Recently, different conceptual and analytical frameworks have been applied to translational neuroscience studies of the neural substrates of the MMN. Two critical subcomponent processes have been identified as contributing to MMN generation: (1) adaptation to frequently presented stimuli and (2) deviance detection in response to infrequent, oddball stimuli. The stimulus-specific adaptation (SSA) hypothesis suggests that upon being presented with a repetitive auditory stimulus, neurons in the auditory cortex show a specific decrease in their response.^18^ Neural adaptation should occur more strongly to standard repetitive stimuli than deviant stimuli that are rarely presented in an oddball paradigm (figure 1). This “adaptation hypothesis” of the MMN is related to differences between the deviant stimuli and adapted neural responses to standard stimuli.^19^ Animal studies demonstrate that adaptation components contribute to the MMN in the primary auditory cortex (A1).^15,18,20^ In contrast, the “deviance detection hypothesis” posits that responses to infrequent oddball stimuli are dissociable from responses to standard stimuli, with mismatch response amplitudes being determined by both tone differences and deviant probability. Since these competing hypotheses about the nature of the MMN cannot be disentangled by the conventional auditory oddball paradigm, the “many-standards paradigm” was first developed by Jacobsen et al in healthy volunteers.^21–23^ This paradigm (figure 1) comprehensively controls for important stimulus features that are embedded within conventional auditory oddball paradigms, allowing for the distinct assessment of (1) adaptation to frequently presented stimuli (adaptation component), (2) deviant probability (deviance detection component), and (3) responses to different tones when matched on their probabilities (tone difference component).

To our knowledge, no previous studies have used the many-standards paradigm in patients with schizophrenia to show the relative contributions of adaptation, tone difference, and deviance detection to reduced MMN in schizophrenia. Previous animal studies have attempted to deepen the understanding of the molecular mechanisms underlying adaptation and deviance detection in the MMN using the many-standards paradigm.^24,25^ Farley et al investigated the MMN in the auditory cortex of rats and found that an MMN-like response in the A1 region was due to SSA and was not altered by the administration of NMDA receptor antagonists.^20^ Fishman and Steinschneider measured the MMN in the auditory cortex of monkeys and reported similar findings.^15^ In contrast, several studies have reported a deviance detection component in mice and rats.^14,26–29^ In rats, the deviance detection component could be altered by the administration of NMDA receptor antagonists.^28,29^ These findings suggest that deviance detection may depend on functional NMDA receptors, and a clinical study performing the same paradigm in patients with schizophrenia may help better understand the pathophysiology reflected by MMN reduction.

Based on this collective pattern of findings, we predicted that the deviance detection component would be selectively altered in schizophrenia. In this study, we used the classic auditory oddball paradigm and the many-standards paradigm and obtained electroencephalographic data from groups of patients with schizophrenia and healthy comparison subjects (HCS) to investigate whether a reduced MMN reflects an impairment in adaptation or deviance detection in patients with schizophrenia. Because previous meta-analyses^2,3^ have shown differences in the magnitude of MMN impairment in patients with schizophrenia when responses are elicited to stimuli that differ in their duration (dMMN) vs frequency (fMMN), both conditions were assessed in this study.

## Methods

### Subjects

Twenty-five patients with schizophrenia and 27 HCS participated in this study (table 1, Supplementary Method 1). Written informed consent was obtained from each subject. The Research Ethics Committee of the Faculty of Medicine at the University of Tokyo approved this study (No.629). For participants under 20 years old, we obtained written informed consent both from the participants and the parents of the participants.

**Table 1.** Comparisons of Patients With Schizophrenia and Healthy Comparison Subjects

	Patients With Schizophrenia	Healthy Comparison Subjects	Statistics
*N* (sex ratio M/F)^a^	25 (14/11)	27 (11/16)	*χ* ^2^ = 1.21, *df* = 1, *P* = .27
Age (y)^b^	31.7 (10.4)	32.5 (7.5)	*t*_50_ = −0.32, *P* = .75
Education (y)^b^	14.1 (2.5)	18.3 (2.6)	*t*_50_ = −5.95, *P* < .001*
DOI (y)	10.8 (8.2)		
PANSS			
Positive	17.4 (4.9)		
Negative	21.0 (6.1)		
General	37.2 (8.5)		
Total	75.6 (18.0)		
GAF-S score	47.9 (10.4)		
GAF-F score	50.5 (10.2)		
Antipsychotic dose (mg/day)	646.3 (434.0)		
Duration MMN	–0.95 (0.49)	–1.64 (1.07)	*d* = 0.83
Adaptation	–0.66 (0.73)	–0.72 (0.68)	*d* = 0.08
Tone difference	–0.42 (0.75)	–0.60 (0.73)	*d* = –0.24
Deviance detection	–0.71 (0.74)	–1.52 (1.10)	*d* = 0.86
Frequency MMN	–0.32 (0.52)	–0.90 (0.75)	*d* = 0.90
Adaptation	0.45 (0.52)	0.41 (0.50)	*d* = 0.07
Tone difference	–0.13 (0.53)	–0.20 (0.59)	*d* = 0.13
Deviance detection	–0.64 (0.52)	–1.11 (0.83)	*d* = 0.68

*Note*: DOI, duration of illness; PANSS, Positive and Negative Syndrome Scale; GAF-S, Global Assessment of Functioning-Symptom; GAF-F, Global Assessment of Functioning-Functioning; MMN, mismatch negativity.

All values are shown as the mean (standard deviation). *d* indicates Cohen’s *d* effect size; the antipsychotics were converted to a chlorpromazine-equivalent dose.

^a^Chi-square test.

^b^Independent *t*-test.

*P < .05.

### Oddball Paradigm

We used a 2-tone auditory oddball paradigm with 2000 stimuli to assess the dMMN. Standard tones (1000 Hz, 50 ms) comprised 90% of the stimuli, and deviant tones (1000 Hz, 100 ms) comprised 10% of the stimuli (figure 1A). We used a different 2-tone auditory oddball paradigm with 2000 stimuli to assess the fMMN; standard tones (1000 Hz, 50 ms) comprised 90% of the stimuli, and deviant tones (1200 Hz, 50 ms) comprised 10% of the stimuli (figure 1B). The details are provided in Supplementary Method 2.

### Many-Standards Paradigm

We used the many-standards paradigm as a control task for the oddball paradigm, consistent with previous studies.^29^ In the many-standards paradigm utilizing different durations, 10 tones with different durations (10, 25, 50, 75, 100, 125, 150, 175, 200, and 225 ms; 1000 Hz; 2000 stimuli in total) were presented with an equal probability of 10% (figure 1A). In the many-standards paradigm utilizing different frequencies, 10 tones with different frequencies (700, 800, 900, 1000, 1100, 1200, 1300, 1400, 1500, and 1600 Hz; 50 ms; 2000 stimuli in total) were presented with an equal probability of 10% (figure 1B). Combining the many-standards paradigm and the classic oddball paradigm enabled us to divide the MMN into 3 components: adaptation, tone difference, and deviance detection (figure 1).

### Mismatch Negativity

The MMN can be calculated by comparing event-related potentials (ERPs) in response to deviant stimuli (Deviant 100 ms or Deviant 1200 Hz) and in response to standard stimuli (Standard 50 ms or Standard 1000 Hz) using only the oddball paradigm.

$$\begin{array}{l} \begin{array}{ll} & dMMN=Deviant\;100\;ms\;-\;Standard\;50\;ms\\ & fMMN=Deviant\;1200\;Hz\;-\;Standard\;1000\;Hz \end{array} \end{array}$$

Furthermore, we divided the MMN into 3 components: adaptation, tone difference, and deviance detection using 2 paradigms: the oddball paradigm and the many-standards paradigm (figure 1).

### Adaptation Component

The adaptation component indicates that neural activity shows a decreased response to auditory stimuli after repetitive presentations. Because standard stimuli are repetitively presented in the oddball paradigm, neural adaptation affects the ERPs in response to standard stimuli (Standard 50 ms or Standard 1000 Hz). In contrast, because the identical tone as that of the standard stimulus in the oddball paradigm (control stimulus) is rarely presented in the many-standards paradigm, neural adaptation has little effect on the ERPs in response to control stimuli (Control 50 ms or Control 1000 Hz) in this paradigm. Neural adaptation can be evaluated by comparing ERPs in response to standard stimuli in the oddball paradigm with ERPs in response to control stimuli in the many-standards paradigm (Control 50 ms vs Standard 50 ms or Control 1000 Hz vs Standard 1000 Hz).

$$\begin{array}{l} \begin{array}{ll} & Adaptation\;component\;for\;dMMN\\ & \;=Control\;50\;ms\;-\;Standard\;50\;ms\\ & Adaptation\;component\;for\;fMMN\\ & \;=Control\;1000\;Hz\;-\;Standard\;1000\;Hz \end{array} \end{array}$$

### Tone Difference Component

Since deviant stimulus tones (Deviant 100 ms or Deviant 1200 Hz) were different from standard stimulus tones (Standard 50 ms or Standard 1000 Hz) in the oddball paradigm, the effect of the tone difference on the MMN must be considered in addition to adaptation and deviance detection. The pair of tones was simply different in their duration or frequency, and those effects need to be examined without the background of repetitive or rare stimuli. The tone difference can be calculated by comparing the same tones in the many-standards paradigm, which has no repetitive or rare stimuli (Control 50 ms vs Control 100 ms or Control 1000 Hz vs Control 1200 Hz).

$$\begin{array}{l} \begin{array}{ll} & Tone\;dif\;ference\;component\;for\;dMMN\\ & \;=Control\;100\;ms\;-\;Control\;50\;ms\\ & Tone\;dif\;ference\;component\;for\;fMMN\\ & \;=Control\;1200\;Hz\;-\;Control\;1000\;Hz \end{array} \end{array}$$

### Deviance Detection Component

Deviance detection indicates the neural process that detects deviance based on the context of the auditory stimuli. In the oddball paradigm, deviant stimuli (Deviant 100 ms or Deviant 1200 Hz) are, by definition, detected as deviant because they are rarely presented, whereas standard stimuli are repetitively presented. In contrast, in the many-standards paradigm, there is no deviance because each tone (Control 100 ms or Control 1200 Hz) is presented with equal probability. We can evaluate the effect of the deviance detection component by comparing the ERPs in response to deviant stimuli in the oddball paradigm with the ERPs in response to the identical tone in the many-standards paradigm (Deviant 100 ms vs Control 100 ms or Deviant 1200 Hz vs Control 1200 Hz).

$$\begin{array}{l} \begin{array}{ll} & Deviance\;detection\;component\;for\;dMMN\\ & \;=Deviant\;100\;ms\;-\;Control\;100\;ms\\ & Deviance\;detection\;component\;for\;fMMN\\ & \;=Deviant\;1200\;Hz\;-\;Control\;1200\;Hz \end{array} \end{array}$$

### Statistical Analysis

We employed *χ*^*2*^ tests and independent *t-*tests to compare demographics and clinical characteristics between the schizophrenia and HCS groups. We performed a repeated measures analysis of variance (ANOVA) with pairs of stimuli (eg, Standard 50 ms and Control 50 ms) as the within-subject factor and with the 2 groups (patients with schizophrenia and HCS) as the between-subjects factor to examine the MMN, adaptation, tone differences, and deviance detection. If we obtained a significant main effect of the stimulus, it would indicate that the component significantly consisted of MMN over the groups. If the interaction between the stimulus and the group was significant, it would indicate that the difference in the components between the groups (patients with schizophrenia and HCS) was significant. The significance level was set at *P* < .00625 (.05/8) adjusted with Bonferroni correction for repeated measures ANOVA. Cohen’s *d* effect sizes were calculated from the overall group contrast to compare the MMN and each component of the MMN. If we found a significant interaction in repeated measures ANOVA, post hoc analyses were performed with paired *t-*tests within each group and independent *t-*tests between the groups.

For supplementary information, the Pearson correlation coefficients (*r*) of the MMN amplitude or the components of the MMN amplitude with Positive and Negative Syndrome Scale (PANSS) scores or Global Assessment of Functioning (GAF) scores in the schizophrenia group were calculated (supplementary results 1 and supplementary table 1). In addition, we analyzed how adaptation or deviance detection affects the P3a component (supplementary methods 2 and supplementary results 2). The significance level was set at *P* < .05 adjusted with Bonferroni correction.

## Results

### Mismatch Negativity

**Fig. 2.**
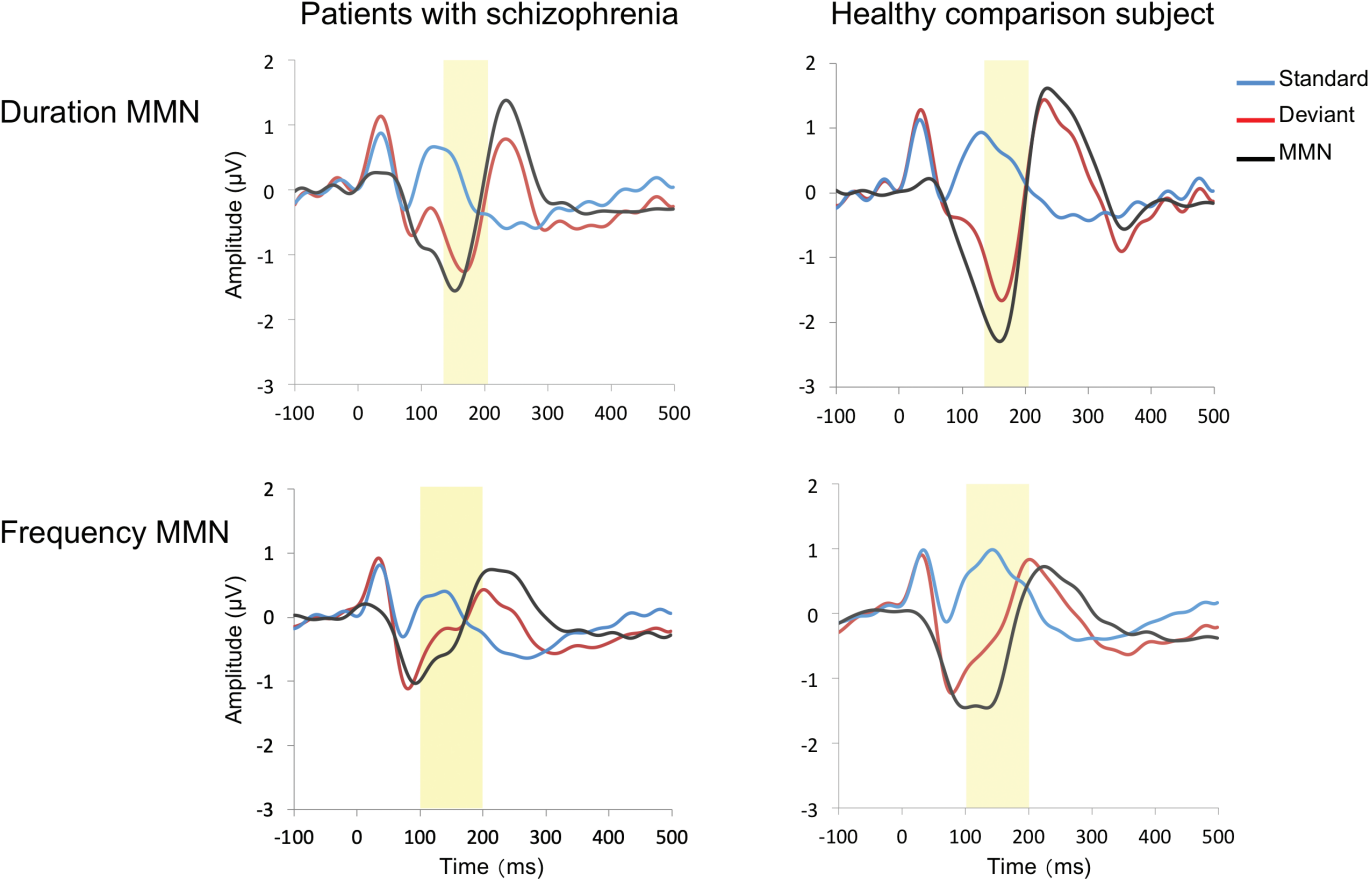
The average waveforms of mismatch negativity (MMN) at the FCz in patients with schizophrenia and healthy comparison subjects. The amplitude of the duration MMN was measured as the mean voltage from 135 to 205 ms post-stimulus, and the amplitude of the frequency MMN was measured as the mean voltage from 100 to 200 ms (shaded yellow) (Colored figure is available online).

**Fig. 3.**
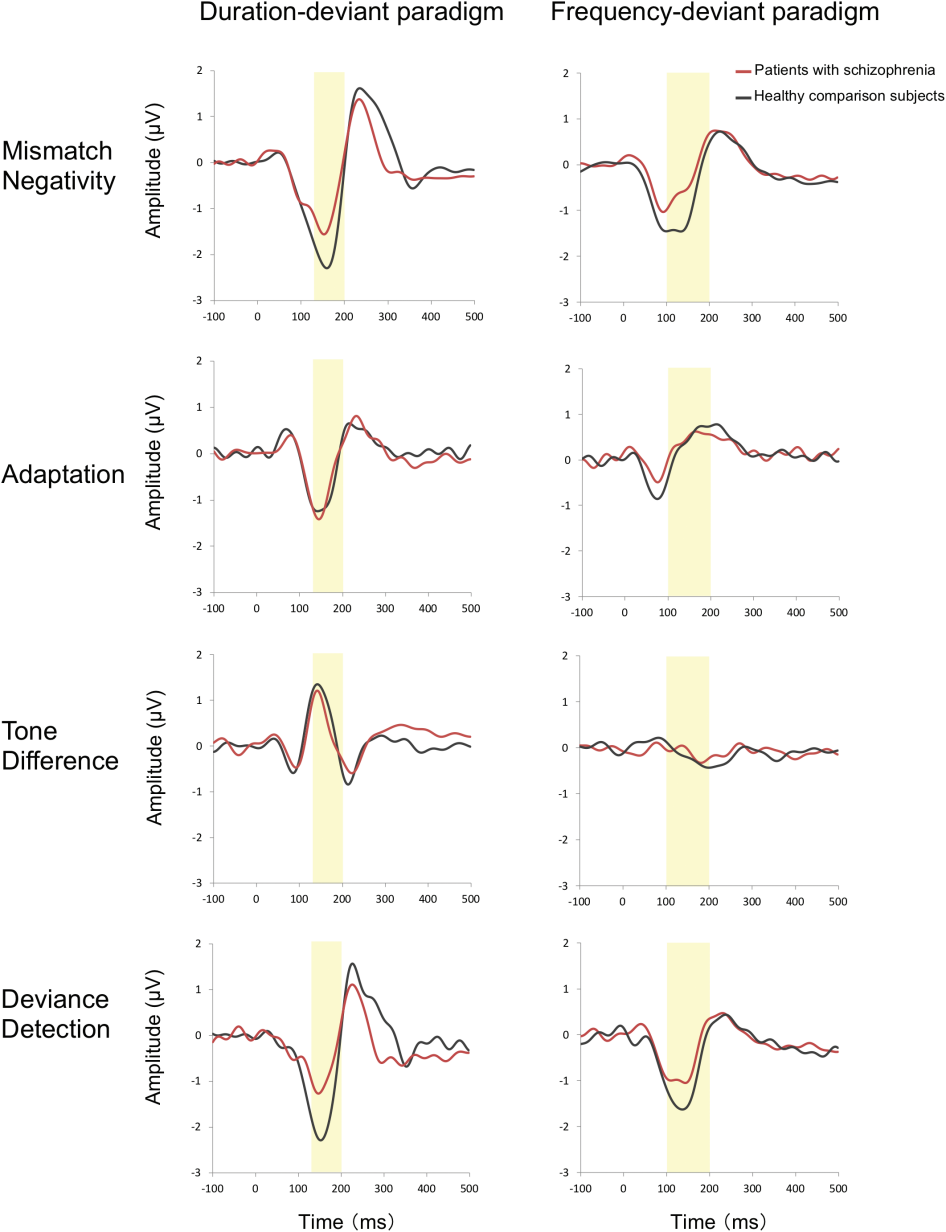
The average waveforms for mismatch negativity (MMN) and each component of the MMN at the FCz (Colored figure is available online).

The average waveforms for standard stimuli and deviant stimuli are shown in figure 2. The grand average waveforms for the MMNs in patients and HCS are shown in figure 3. The topographies of the MMN amplitudes are shown in supplementary figure 1. In the analysis of the dMMN, repeated measures ANOVA showed a significant main effect of stimulus (*F*_1, 50_ = 122.5, *P* < .001) and an interaction between stimulus and group (*F*_1, 50_ = 8.8, *P* = .005), but no significant main effect of group (*F*_1, 50_ = 0.57, *P* = .45). Thus, the amplitude of the dMMN was significantly reduced in patients compared with that in HCS (*t*_50_ = 2.96, *P* = .005, *d* = 0.83; table 1).

In the analysis of the fMMN, repeated measures ANOVA showed a significant main effect of stimulus (*F*_1, 50_ = 45.5, *P* < .001) and an interaction between stimulus and group (*F*_1, 50_ = 10.3, *P* = .002), but no significant main effect of group (*F*_1, 50_ = 3.1, *P* = .08). Thus, the amplitude of the fMMN was significantly reduced in patients compared with that in HCS (*t*_50_ = 3.21, *P* = .002, *d* = 0.90).

### Adaptation Component

**Fig. 4.**
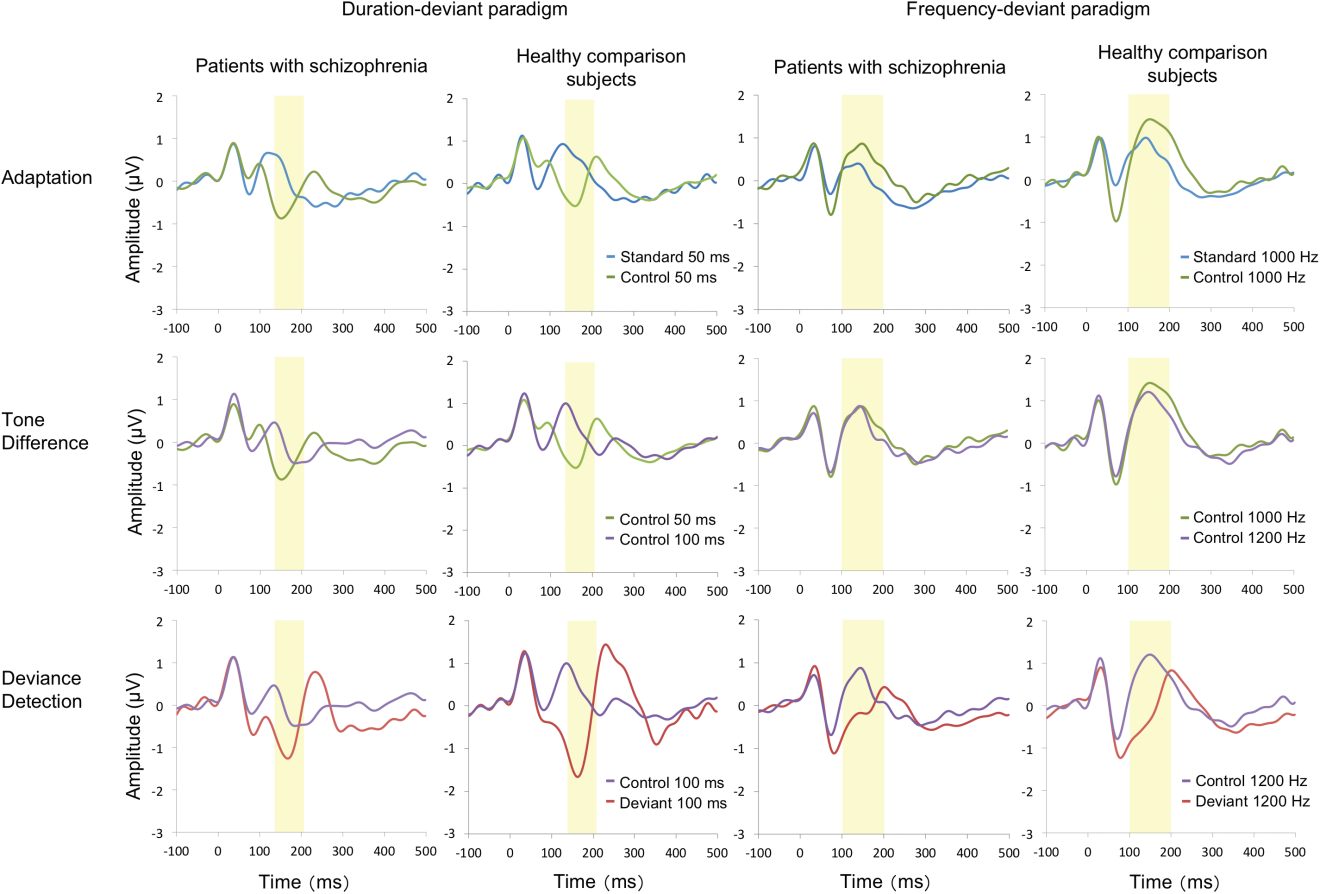
The average waveforms at the FCz in the oddball paradigm and many-standards paradigm (Colored figure is available online).

The average waveforms for each stimulus are shown in figure 4. The grand average waveforms for each component are shown in figure 3. In the analysis of the dMMN adaptation component, repeated measures ANOVA showed a significant main effect of stimulus (*F*_1, 50_ = 49.6, *P* < .001) but no significant main effect of group (*F*_1, 50_ = 8.1, *P* = .0064) or interaction between stimulus and group (*F*_1, 50_ = 0.08, *P* = .78). Thus, there was a significant adaptation component in both groups that contributes to dMMN, but there was no significant difference in the adaptation component of the dMMN amplitude between patients and HCS (*d* = 0.08)

In the analysis of the fMMN adaptation component, repeated measures ANOVA showed a significant main effect of stimulus (*F*_1, 50_ = 36.9, *P* < .001) and a main effect of group (*F*_1, 50_ = 14.8, *P* < .001), but no interaction between the stimulus and group (*F*_1, 50_= 0.07, *P* = .79). Therefore, there was a significant adaptation component in both groups that contributes to fMMN. However, there was no significant difference in the adaptation component of the fMMN between patients and HCS (*d* = 0.07). Inspecting figure 2, it appears that, for the fMMN, the amplitudes in response to the deviant tone are similar across groups, while the amplitudes in response to the standard tone are larger in HCS than patients. In post hoc analyses, the response to the deviant tone was not significantly different between the groups (*t*_50_ = 0.13, *P* = .90); however, the response to the standard tone was significantly different between the groups (*t*_50_ = –4.2, *P* < .001).

### Tone Difference Component

In the analysis of the dMMN tone difference component, repeated measures ANOVA showed a significant main effect of stimulus (*F*_1, 50_ = 24.9, *P* < .001) and a main effect of the group (*F*_1, 50_ = 9.1, *P* = .004), but no interaction between stimulus and group (*F*_1, 50_ = 0.72, *P* = .40). Thus, there was a significant tone difference component in both groups that contributes to dMMN, but there was no significant difference in the tone difference component of the dMMN amplitude between patients and HCS (*d* = –0.24).

In the analysis of the fMMN tone difference component, repeated measures ANOVA showed no significant main effect of stimulus (*F*_1, 50_ = 4.4, *P* = .04), main effect of group (*F*_1, 50_= 8.0, *P* = .007) or interaction between stimulus and group (*F*_1, 50_ = 0.22, *P* = 0.64). Therefore, there was no significant tone difference component in either group that contributes to fMMN, and there was no significant difference in the tone difference component of the fMMN amplitude between patients and HCS (*d* = 0.13).

### Deviance Detection Component

In the analysis of the dMMN deviance detection component, repeated measures ANOVA showed a significant main effect of stimulus (*F*_1, 50_ = 71.9, *P* < .001) and an interaction between stimulus and group (*F*_1, 50_ = 9.5, *P* = .003), but no significant main effect of group (*F*_1, 50_ = 1.1, *P* = .30). Post hoc *t*-tests revealed significant differences between the Deviant 100 ms and Control 100 ms stimuli in patients (*t*_24_ = –4.78, *P* < .001) and HCS (*t*_26_ = –7.16, *P* < .001); thus, there was a significant deviance detection component in both groups that contributes to dMMN, and there was a significant difference in the deviance detection component of the dMMN amplitude between patients and HCS (*t*_50_ = 3.08, *P* = .003, *d* = 0.86).

In the analysis of the fMMN deviance detection component, repeated measures ANOVA showed a significant main effect of stimulus (*F*_1, 50_ = 82.2, *P* < .001) but no significant main effect of group (*F*_1, 50_ = 1.4, *P* = .25) or interaction between stimulus and group (*F*_1, 50_ = 5.9, *P* = .02). Therefore, there was a significant deviance detection component in both groups that contributes to fMMN, but there was no significant difference in the deviance detection component of the fMMN amplitude between patients and HCS (*d* = 0.68).

## Discussion

This study comprehensively controlled for important stimulus characteristics associated with MMN studies and resolved longstanding questions about the nature of MMN impairment in schizophrenia. Patients with schizophrenia showed dMMN and fMMN deficits with a large effect size when measured via a conventional auditory oddball paradigm, consistent with the results of many previous studies.^2,3^ Importantly, the many-standards paradigm confirmed selective impairments in deviance detection with normal adaptation to frequently presented stimuli (adaptation component) and responses to tones with different physical characteristics when matched on deviant probability (tone difference component) in patients with schizophrenia.

Our findings are consistent with those of previous studies that have relied on different paradigms for MMN elicitation. For example, Coffman et al have shown impairment in the MMN but not repetition suppression in patients with schizophrenia using an oddball paradigm with complex deviants^30^; McCleery et al have shown attenuated prediction error processing in patients with schizophrenia using a roving standard paradigm.^31^ However, these prior studies examined prediction error using complicated paradigms that do not directly relate to the stimulus features of a conventional auditory oddball paradigm.^30–32^ Moreover, such paradigms may lead to the underestimation of the effect sizes of schizophrenia-related impairments.^33^ Because we focused on MMN reduction assessed by the classic oddball paradigm using simple deviants, which is the most common paradigm that has been used in previous studies in patients with schizophrenia,^33^ we divided the MMN into the components using both an oddball paradigms using simple deviants and the many-standards paradigm. This method has already been applied to animal model studies^15,20,24–29^ and an electrocorticography (ECoG) study^34^ aiming to clarify the location and related molecular mechanism of deviance detection components; however, to our knowledge, there have been no studies performed in patients with schizophrenia.

Our findings indicate that a reduction in the MMN amplitude in patients with schizophrenia reflects a selective impairment in deviance detection. This finding is consistent with NMDA receptor involvement in schizophrenia because clinical studies have shown that the reduced MMN amplitude can reflect alterations in glutamatergic neurotransmission, and concordant animal studies have shown that deviance detection depends on NMDA receptor function.^10,11,28,29,35–38^ Furthermore, Ishishita et al recently found that deviance detection, but not adaptation, was the dominant component of the MMN in the lateral superior temporal gyrus (STG) in their ECoG study using the many-standards paradigm.^34^ Thus, these results imply that the disruption of deviance detection in the STG underlies MMN reduction and that this disruption may reflect the dysfunction of NMDA receptor function in patients with schizophrenia.

Recent animal studies have been trying to interpret the MMN in terms of predictive coding models of brain function, which were shown by computational model studies.^39^ In their framework, Malmierca and colleagues refer to deviance detection as predictive error and adaptation as repetition suppression and have shown the neural basis of prediction error along the auditory pathway from the subcortical level to the cortex in rodent models.^40,41^ Our findings may help promote animal model studies using these frameworks to reveal the pathophysiology of schizophrenia.

This study has some limitations. First, the sample sizes were modest in this study. However, according to power analysis, the sample size of this study is an adequate sample size. The previous meta-analysis of MMN indicates that the Cohen’s *d* effect size for the duration MMN is 0.94; the power analysis (*t*-test, alpha = 0.05, power = 0.80, 2-tailed) shows that the sample size needed for each group is *N* = 19.^3^ Second, all patients were treated with antipsychotic medications at the time of testing. Although previous large-scale studies failed to demonstrate a robust effect of antipsychotic medications on the MMN in patients with schizophrenia using a conventional auditory oddball paradigm,^42,43^ the effect of medication on specific adaptations, deviance detection, and tone differences extracted from the many-standards paradigm cannot be completely ruled out. Nonetheless, no significant correlations were detected between chlorpromazine equivalents and components of the MMN in patients (–0.22 < *r* < 0.37, *P* > .07). Third, the patients in this study were in the chronic stages of the disease, and, therefore, the effects of each component on the MMN may differ between the chronic and early stages. Future studies on patients in the earlier stages are required to clarify and generalize the stage-specific effects of each component on the MMN. Fourth, in the many-standards paradigm, there is an intervening sound, ie, Standard 75 ms in the case of Control 100 ms and Control 50 ms and Standard 1100 Hz in the case of Control 1200 Hz and Control 1000 Hz (figure 1). There may be both frequency- and duration-tuned neurons in the auditory cortex. It is possible that there is greater cross-adaptation of responses to 50 ms and 100 ms duration or 1000 Hz and 1200 Hz frequencies in the many-standards paradigm than in the oddball paradigm, which could result in smaller event-related potentials to the control sounds and may lead to an overestimation of deviance detection and an underestimation of repetitive adaptation effects. Fifth, in this study, electrodes were referenced to the vertex, and the EEG data were re-referenced to an average reference. Mastoids or nose references are widely used to measure the MMN in clinical research. Therefore, comparing the results of this study with those of previous studies needs to be assessed carefully.

In conclusion, we found that the deviance detection component of the auditory MMN was reduced in patients with schizophrenia compared with that in HCS. The segregation and clarification of the neural mechanisms of deviance detection and the adaptation component in both clinical and animal studies will strengthen the utility of the MMN as a translatable brain marker, which should further pave the way for clarifying the pathophysiology of schizophrenia and the development of novel interventions.

## Supplementary Material

sbaa006_suppl_Supplementary_Information

sbaa006_suppl_Supplementary_Figure_1

sbaa006_suppl_Supplementary_Table_1
