# Supplementary material for: Reduced Auditory Mismatch Negativity Reflects Impaired Deviance Detection in Schizophrenia
Source: Schizophr Bull. 2020 Feb 19;46(4):937–46. doi: 10.1093/schbul/sbaa006 (PMC7345817; doi:10.1093/schbul/sbaa006)
Supplement: sbaa006_suppl_Supplementary_Information [file sbaa006_suppl_supplementary_information.docx]

**Supplementary Information**

**Reduced auditory mismatch negativity reflects impaired deviance detection in schizophrenia**

Daisuke Koshiyama, M.D., Ph.D.^1^; Kenji Kirihara, M.D., Ph.D.^1^; Mariko Tada, M.D., Ph.D.^1, 2^; Tatsuya Nagai, M.D., Ph.D.^1, 3^; Mao Fujioka, M.D.^1^; Kaori Usui, M.A.^1^; Tsuyoshi Araki, M.D., Ph.D.^1^; Kiyoto Kasai M.D., Ph.D.^1, 2^

1. Department of Neuropsychiatry, Graduate School of Medicine, The University of Tokyo, Tokyo, Japan
2. The International Research Center for Neurointelligence (WPI-IRCN) at The University of Tokyo Institutes for Advanced Study (UTIAS), The University of Tokyo, Tokyo, Japan
3. Department of Psychiatry, Kawamuro Memorial Hospital, Niigata, Japan

**TABLE OF CONTENTS**

SUPPLEMENTARY METHODS

Supplementary Method 1 Subjects…...……...……………………….…….....2

Supplementary Method 2 Electroencephalography recordings and analyses…2

SUPPLEMENTARY RESULTS

Supplementary Result 1 The results of correlations with clinical symptoms....3

Supplementary Result 2 The results of the P3a component….................….....4

SUPPLEMENTARY REFERENCES..................………….................................…………………….….….....6

**SUPPLEMENTARY METHODS**

**Supplementary Method 1** Subjects

Patients with schizophrenia were recruited from outpatient and inpatient units at the University of Tokyo Hospital. Healthy comparison subjects were recruited through advertisements on a social media network, and some of the comparison subjects were medical staff of the University of Tokyo. Inclusion criteria for patients with schizophrenia were a diagnosis of schizophrenia using the Diagnostic and Statistical Manual of Mental Disorders, Fourth Edition (DSM-IV) and age of 15–50 years old. Inclusion criteria for healthy comparison subjects were age 15–50 years old and no personal history of psychiatric disease or a family history of schizophrenia in first-degree relatives. Exclusion criteria for all groups were neurological illness; traumatic brain injury with a loss of consciousness for more than five minutes; a low premorbid intelligence quotient (IQ; below 70); previous alcohol/substance abuse or addiction; and hearing impairment, as assessed by an audiometer hearing test in both ears with a 30-dB sound pressure level tone at 1000 Hz and with a 40-dB at 4000 Hz. The participants abstained from coffee and alcohol on the EEG measurement day. The Positive and Negative Syndrome Scale (PANSS) and the Global Assessment of Functioning (GAF) were used to assess global functioning and clinical symptoms in all participants with schizophrenia.^1-3^ All patients took antipsychotic medication. The antipsychotics were converted to a chlorpromazine-equivalent dose.^4^

**Supplementary Method 2** Electroencephalography recordings and analyses

We performed the 4 paradigms on the same day. Each paradigm took approximately 15 minutes. The interval each paradigm was several minutes. The 4 paradigms were counterbalanced using 2 patterns: the 1st order was dMMN oddball, dMMN many-standards, fMMN oddball and fMMN many-standards paradigm; and the 2nd order was fMMN many-standards, fMMN oddball, dMMN many-standards and dMMN oddball paradigm.

A 64-channel Geodesic EEG System (Electrical Geodesics Inc., Eugene, OR) was used to obtain EEG data. Electrodes were referenced to the vertex, and impedances were kept below 50 kΩ. The sampling rate was 500 Hz. All of the stimuli were 80 dB and had a 1-ms rise/fall time. The stimulus onset asynchrony was 500 ms. Paradigms were counterbalanced. While the participants watched a silent cartoon, the tones were presented binaurally through inserted earphones. The EEG data were analyzed using EEGLAB.^5^ The continuous EEG data were digitally filtered at 1–20 Hz and segmented from −100 to 500 ms relative to stimulus onset. Independent component analysis was used for eye blink correction. Epochs exceeding ±100 μV at any electrode were rejected. The EEG data were rereferenced to an average reference. The mean of the prestimulus baseline was subtracted for baseline correction. After averaging across trials, the amplitude at FCz was used for further analyses.

For analyses in paradigms with duration deviants, we measured the mean amplitude of mismatch negativity (MMN) from 135 to 205 ms post stimulus, in accordance with previous studies.^6-8^ For analyses in paradigms with frequency deviants, we measured the mean amplitude of the MMN from 100 to 200 ms, in accordance with previous studies.^6, 9, 10^

In the analyses for the P3a component, we first identified the peak of the duration P3a (dP3a, response to duration deviants) and the frequency P3a (fP3a, response to frequency deviants) with a latency window of 250 to 400 ms poststimulus and then calculated the mean amplitudes of dP3a and fP3a over 50-ms windows surrounding the identified peaks according to a previous study.^6^

**SUPPLEMENTARY RESULTS**

**Supplementary Result 1** The results of correlations with clinical symptoms

There were no significant correlations between the MMN amplitude or the components of the MMN amplitude and the PANSS scores or GAF scores in the schizophrenia group at a significance level of *p*< 0.001 (0.05/40), adjusted with the Bonferroni correction (**Supplementary Table 1**). PANSS positive scores were correlated with the dMMN amplitude (*r*= 0.46, *p*= 0.02) and the deviance detection (*r*= 0.42, *p*= 0.04) and tone difference (*r*= 0.41, *p*= 0.04) components of the dMMN amplitude; PANSS negative scores were correlated with the fMMN amplitude (*r*= –0.45, *p*= 0.02); the PANSS general score was correlated with the tone difference component of the dMMN amplitude (*r*= 0.45, *p*= 0.02) in patients at an uncorrected *p*-value level of < 0.05.

**Supplementary Result 2** The results of the P3a component

*P3a component*

In the analysis of dP3a, repeated-measures ANOVA showed a significant main effect of stimulus (*F*_1, 50_= 73.6, *p*< 0.001) but no significant main effect of group (*F*_1, 50_= 2.8, *p*= 0.10) or interaction between stimulus and group (*F*_1, 50_= 8.0, *p*= 0.007). The significance level was set at *p*< 0.00625 (0.05/8) adjusted with Bonferroni correction for repeated-measures ANOVA. Thus, the amplitude of the dP3a was not significantly different between patients [mean, 1.06; standard deviation (SD), 0.85] and HCS (mean, 1.57; SD, 1.30; *d*= –0.46).

In the analysis of the fP3a adaptation component, repeated-measures ANOVA showed a significant main effect of stimulus (*F*_1, 50_= 47.2, *p*< 0.001) but no significant main effect of group (*F*_1, 50_= 1.1, *p*= 0.29) or interaction between stimulus and group (*F*_1, 50_= 0.04, *p*= 0.84). Therefore, the amplitude of the fP3a was not significantly different between patients (mean, 0.66; SD, 0.69) and HCS (mean, 0.62; SD, 0.66; *t*_50_= 0.25, *p*= 0.80, *d*= 0.05).

*Adaptation component of the P3a component*

In the analysis of the dP3a adaptation component, repeated-measures ANOVA showed a significant main effect of stimulus (*F*_1, 50_= 26.8, *p*< 0.001) but no significant main effect of group (*F*_1, 50_= 2.5, *p*= 0.12) or interaction between stimulus and group (*F*_1, 50_= 0.14, *p*= 0.71). Thus, the adaptation component significantly consists of dP3a over the groups, but there was no significant difference in the adaptation component of the dP3a amplitude between patients (mean, 0.52; SD, 0.78) and HCS (mean, 0.45; SD, 0.55; *d*= 0.10).

In the analysis of the fP3a adaptation component, repeated-measures ANOVA showed a significant main effect of stimulus (*F*_1, 50_= 28.5, *p*< 0.001) but no significant main effect of group (*F*_1, 50_= 2.3, *p*= 0.14) or interaction between stimulus and group (*F*_1, 50_= 0.0002, *p*= 0.99). Therefore, the adaptation component significantly consists of fP3a over the groups, but there was no significant difference in the adaptation component of fP3a between patients (mean, 0.37; SD, 0.51) and HCS (mean, 0.38; SD, 0.50; *d*= –0.004).

*Tone difference component of the P3a component*

In the analysis of the dP3a tone difference component, repeated-measures ANOVA showed no significant main effect of stimulus (*F*_1, 50_= 0.02, *p*= 0.88), main effect of group (*F*_1, 50_= 1.0, *p*= 0.31), or interaction between stimulus and group (*F*_1, 50_= 0.04, *p*= 0.84). Thus, the tone difference component does not significantly consist of dP3a over the groups. There was no significant difference in the tone difference component of the dP3a amplitude between patients (mean, –0.004; SD, 0.78) and HCS (mean, 0.03; SD, 0.60; *d*= –0.06).

In the analysis of the fP3a tone difference component, repeated-measures ANOVA showed no significant main effect of stimulus (*F*_1, 50_= 1.4, *p*= 0.24), main effect of group (*F*_1, 50_= 1.3, *p*= 0.25), or interaction between stimulus and group (*F*_1, 50_= 0.04, *p*= 0.85). Thus, the tone difference component does not significantly consist of fP3a over the groups. There was no significant difference in the tone difference component of the fP3a amplitude between patients (mean, –0.08; SD, 0.55) and HCS (mean, –0.11; SD, 0.60; *d*= 0.05).

*Deviance-detection component of the P3a component*

In the analysis of the dP3a deviance-detection component, repeated-measures ANOVA showed a significant main effect of stimulus (*F*_1, 50_= 27.4, *p*< 0.001) but no main effect of group (*F*_1, 50_= 5.3, *p*= 0.03) or interaction between stimulus and group (*F*_1, 50_= 3.0, *p*= 0.09). Thus, the deviance detection component significantly consists of dP3a over the groups, but there was no significant difference in the deviance detection component of the dP3a amplitude between patients (mean, 0.55; SD, 1.14) and HCS (mean, 1.09; SD, 1.12; *d*= –0.48).

In the analysis of the fP3a deviance-detection component, repeated-measures ANOVA showed a significant main effect of stimulus (*F*_1, 50_= 11.5, *p*=0.001), but no significant main effect of group (*F*_1, 50_= 0.76, *p*= 0.39), or interaction between stimulus and group (*F*_1, 50_= 0.001, *p*= 0.97). Thus, the deviance detection component significantly consists of fP3a over the groups, but there was no significant difference in the deviance detection component of the fP3a amplitude between patients (mean, 0.37; SD, 0.76) and HCS (mean, 0.36; SD, 0.78; *d*= 0.01).

**SUPPLEMENTARY REFERENCES**

**1.** Kay SR, Fiszbein A, Opler LA. The positive and negative syndrome scale (PANSS) for schizophrenia. *Schizophr Bull* 1987;13:261-276.

**2.** Hall RC. Global assessment of functioning. A modified scale. *Psychosomatics* 1995;36:267-275.

**3.** Eguchi S, Koike S, Suga M, Takizawa R, Kasai K. Psychological symptom and social functioning subscales of the modified Global Assessment of Functioning scale: reliability and validity of the Japanese version. *Psychiatry Clin Neurosci* 2015;69:126-127.

**4.** Inada T, Inagaki A. Psychotropic dose equivalence in Japan. *Psychiatry Clin Neurosci* 2015;69:440-447.

**5.** Delorme A, Makeig S. EEGLAB: an open source toolbox for analysis of single-trial EEG dynamics including independent component analysis. *J Neurosci Methods* 2004;134:9-21.

**6.** Nagai T, Tada M, Kirihara K, et al. Auditory mismatch negativity and P3a in response to duration and frequency changes in the early stages of psychosis. *Schizophr Res* 2013;150:547-554.

**7.** Michie PT, Innes-Brown H, Todd J, Jablensky AV. Duration mismatch negativity in biological relatives of patients with schizophrenia spectrum disorders. *Biol Psychiatry* 2002;52:749-758.

**8.** Light GA, Braff DL. Mismatch negativity deficits are associated with poor functioning in schizophrenia patients. *Arch Gen Psychiatry* 2005;62:127-136.

**9.** Hirayasu Y, Potts GF, O'Donnell BF, et al. Auditory mismatch negativity in schizophrenia: topographic evaluation with a high-density recording montage. *Am J Psychiatry* 1998;155:1281-1284.

**10.** Salisbury DF, Shenton ME, Griggs CB, Bonner-Jackson A, McCarley RW. Mismatch negativity in chronic schizophrenia and first-episode schizophrenia. *Arch Gen Psychiatry* 2002;59:686-694.
