# Supplementary figures and images for: Reduced Auditory Mismatch Negativity Reflects Impaired Deviance Detection in Schizophrenia
Source: Schizophr Bull. 2020 Feb 19;46(4):937–46. doi: 10.1093/schbul/sbaa006 (PMC7345817; doi:10.1093/schbul/sbaa006)

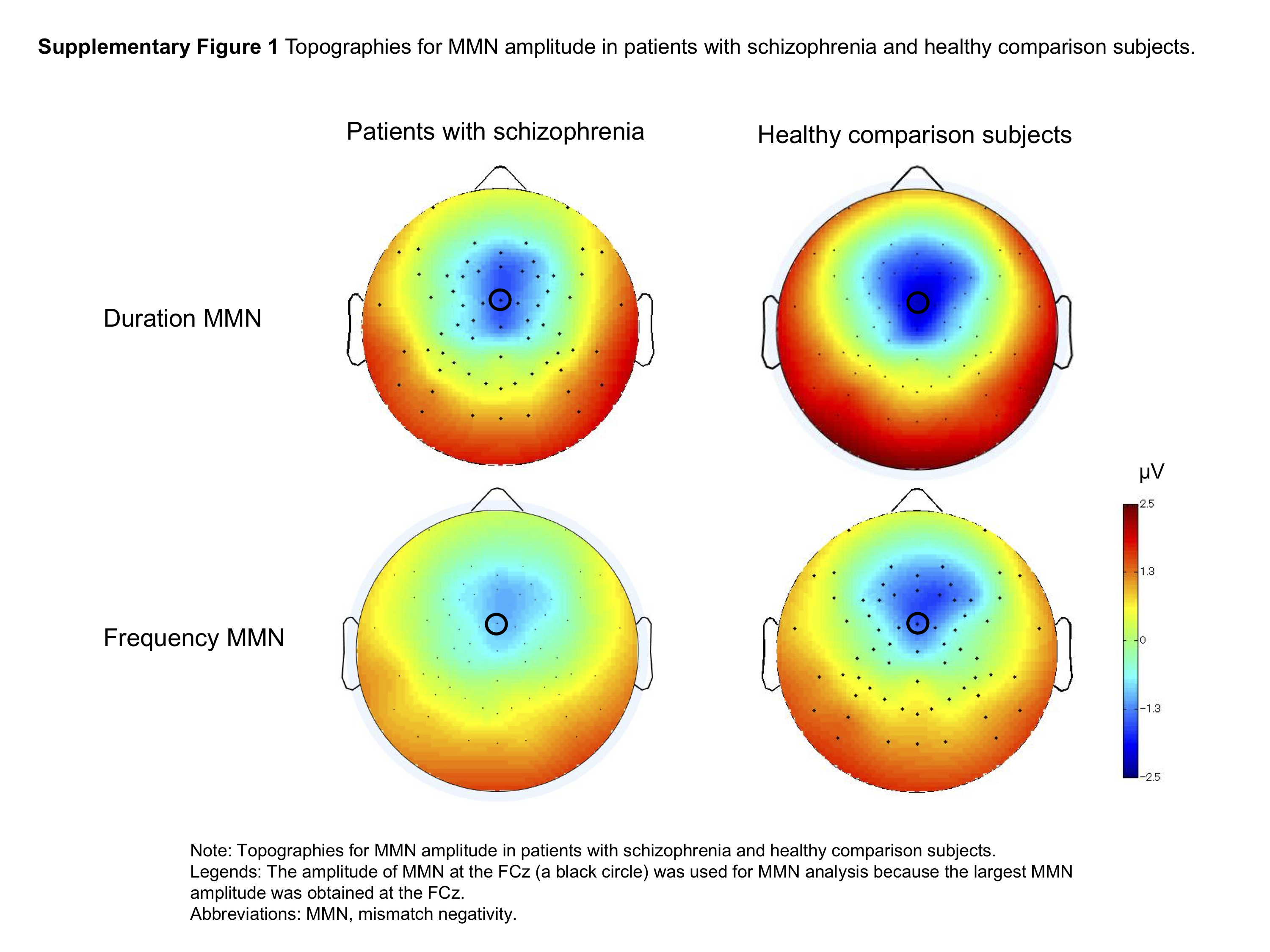

Supplement: sbaa006_suppl_Supplementary_Figure_1 [file sbaa006_suppl_supplementary_figure_1.png]
