# Supplementary material for: Reduced Auditory Mismatch Negativity Reflects Impaired Deviance Detection in Schizophrenia
Source: Schizophr Bull. 2020 Feb 19;46(4):937–46. doi: 10.1093/schbul/sbaa006 (PMC7345817; doi:10.1093/schbul/sbaa006)
Supplement: sbaa006_suppl_Supplementary_Table_1 [file sbaa006_suppl_supplementary_table_1.docx]

**Supplementary Table 1** Correlations of clinical measures with MMN and MMN components in patients with schizophrenia.

|  | PANSS Positive | | PANSS Negative | | PANSS General | | GAF-S score | | GAF-F score | |
| --- | --- | --- | --- | --- | --- | --- | --- | --- | --- | --- |
|  | *r* | *p* | *r* | *p* | *r* | *p* | *r* | *p* | *r* | *p* |
| Duration MMN | 0.46 | 0.02 | 0.06 | 0.77 | 0.39 | 0.06 | –0.25 | 0.22 | –0.09 | 0.68 |
| Adaptation | 0.29 | 0.16 | 0.20 | 0.34 | 0.39 | 0.06 | –0.28 | 0.18 | –0.06 | 0.78 |
| Tone difference | 0.41 | 0.04 | 0.32 | 0.11 | 0.45 | 0.02 | –0.001 | 1.00 | 0.11 | 0.62 |
| Deviance detection | 0.42 | 0.04 | 0.17 | 0.42 | 0.32 | 0.12 | 0.11 | 0.60 | 0.11 | 0.61 |
| Frequency MMN | –0.13 | 0.54 | –0.45 | 0.02 | –0.26 | 0.21 | 0.12 | 0.56 | 0.06 | 0.78 |
| Adaptation | 0.02 | 0.92 | –0.17 | 0.42 | –0.14 | 0.51 | 0.22 | 0.29 | 0.11 | 0.61 |
| Tone difference | –0.12 | 0.56 | 0.09 | 0.66 | –0.01 | 0.95 | –0.30 | 0.14 | –0.33 | 0.10 |
| Deviance detection | –0.03 | 0.89 | –0.38 | 0.06 | –0.11 | 0.60 | 0.21 | 0.32 | 0.29 | 0.16 |

Legend: The significance level was set at *p*<0.00125 (0.05/40) adjusted with the Bonferroni correction.

Abbreviation: PANSS, positive and negative syndrome scale; GAF-S, Global Assessment of Functioning-Symptom; GAF-F, Global Assessment of Functioning-Functioning; MMN, mismatch negativity.
